# Microstructural and Corrosion Properties of Hydroxyapatite Containing PEO Coating Produced on AZ31 Mg Alloy

**DOI:** 10.3390/ma14061531

**Published:** 2021-03-21

**Authors:** Luca Pezzato, Katya Brunelli, Stefano Diodati, Mirko Pigato, Massimiliano Bonesso, Manuele Dabalà

**Affiliations:** 1 Department of Industrial Engineering, University of Padua, Via Marzolo 9, 35131 Padova, Italy; katya.brunelli@unipd.it (K.B.); mirko.pigato@unipd.it (M.P.); massimiliano.bonesso@pd.infn.it (M.B.); manuele.dabala@unipd.it (M.D.); 2 Department of Chemical Science, University of Padua, Via Marzolo 1, 35131 Padova, Italy; stefano.diodati.it@gmail.com; 3 ICMATE-CNR, Corso Stati Uniti 4, 35127 Padova, Italy

**Keywords:** plasma electrolytic oxidation, magnesium alloys, bioabsorbable, corrosion

## Abstract

In this work, the composition of an electrolyte was selected and optimized to induce the formation of hydroxyapatite during Plasma electrolytic oxidation (PEO) treatment on an AZ31 alloy for application in bioabsorbable implants. In detail, the PEO process, called PEO-BIO (Plasma Electrolytic Oxidation-Biocompatible), was performed using a silicate-phosphate-based electrolyte with the addition of calcium oxide in direct-current mode using high current densities and short treatment times. For comparison, a known PEO process for producing anticorrosive coatings, called standard, was applied on the same alloy. The coatings were characterized by scanning electron microscopy (SEM), X-ray diffraction (XRD) and XPS analyses. The corrosion performance was evaluated in simulated body fluid (SBF) at 37 °C. The coating produced on the PEO-BIO sample was porous and thicker than the standard PEO one, with zones enriched in Ca and P. The XRD analysis showed the formation of hydroxyapatite and calcium oxides in addition to magnesium-silicon oxide and magnesium oxide in the PEO-BIO sample. The corrosion resistance of PEO-BIO sample was comparable with that of a traditional PEO treated sample, and higher than that of the untreated alloy.

## 1. Introduction

Magnesium (Mg) alloys are revolutionary biodegradable metals for orthopedic applications thanks to their good biocompatibility, biodegradability, and acceptable mechanical properties. Mg is the fourth most abundant cation in the human body and is essential in many metabolic processes. In detail, Mg is taken daily into the body, stimulates bone cells growth, and accelerates healing [1,2]. The main advantage in the employment of magnesium alloys in the production of implants is that they degrade in vivo due to the presence of Cl^−^ in the physiological environment, thereby eliminating the need for secondary surgeries to remove the implant. The corrosion products (Mg^2+^) are not harmful for the human body [3,4]. Moreover, Mg alloys have mechanical properties (45 GPa of elastic modulus) similar to those of bone (3–20 GPa), unlike titanium alloys and stainless steel (110 and 200 GPa, respectively). Consequently, the stress shielding due to mechanical mismatch between natural bone and metal implants is reduced when Mg alloys are employed [5]. Main competitor materials for bioabsorbable implants are bioceramics and biopolymers. However, bioceramics, such as hydroxyapatite (HAP), are characterized by a brittle nature and by low tensile strength compared to Mg-based alloys, whereas biopolymers are characterized by a low mechanical strength. Therefore, magnesium-based alloys have mechanical properties (density, yield strength, tensile strength, elongation to break and elastic modulus) more similar to that of natural bone compared to other biodegradable alloys, Ti and stainless-steel implants, ceramics and polymers [6,7]. However, magnesium alloys are generally characterized by poor corrosion properties [8]. Magnesium is in fact one of the most active elements and corrodes rapidly in ionic media, such as the human body environment. In the case of implants, controlling the corrosion rate is a key point. In particular, it is necessary for two reasons: first, the implant must possess sufficient strength for the time necessary to allow healing process; secondly, the corrosion rate must be sufficiently slow to not affect the healing process. In fact, while it is true that the by-products of magnesium corrosion are nontoxic, as the metal corrodes, the pH in the localized area increases and this basic environment may impede healing. Similarly, hydrogen gas evolves during the corrosion process and this needs to be reduced [9].

In order to reduce the corrosion rate of the implants in the human body and to promote cell adhesion on the implant surface, a lot of different approaches can be found in the literature. Ding et al. [10] report in a comprehensive review work the effect of the different alloying elements on the corrosion resistance and biocompatibility of Mg alloys. They found that a lot of elements such as Ca, Sr and Zr increase both corrosion resistance and biocompatibility until a certain limit of content. Other elements such as REEs (Rare Earth Elements) are very promising but further studies are needed. Peron et al. [11] report the effect of ECAP (Equal Channel Angular Pressing) on the stress corrosion cracking (SCC) resistance of AZ31 in simulated body fluid (SBF) and found a remarkable decrease in the sensitivity to SCC due to the great grain refinement obtained.

However, generally, in technological applications corrosion properties and biocompatibility are mainly modified through proper surface treatments. Surmaneva et al. [12], for example, reported the production of hydroxyapatite-based coatings on AZ31 magnesium alloy by magnetron sputtering, obtaining coatings with different mechanical properties with the variation of the process parameters. Heimann [13], in his comprehensive work, analyzed different possible treatments for the surface modification of magnesium for biomedical applications. In particular, he considers both chemical and electrochemical conversion coatings, sol-gel coatings, PVD (Physical Vapor Deposition) and RF (Radio Frequency) coatings.

Among the different surface treatments, electrochemical methods are one of the more diffused and among these, Plasma electrolytic oxidation (PEO), also called “Microarc Oxidation (MAO)”, is attracting increasing interest due to the capacity to produce oxide ceramic coatings on light alloys such as Al, Ti and Mg [14,15]. PEO treatment, generally, enhances corrosion- and wear-resistance properties, and can also produce a proper functionalization of the surface [16,17]. PEO is similar to conventional anodizing but is usually carried out in high-voltage condition which is introduced into the high-pressure discharge area from the Faraday region of traditional anodizing. The PEO process produces corona, glow, spark discharge and even microarc discharge phenomena on the surface of the samples. This allows the formation of coatings with oxides containing the elements present in the substrate but also the ones in the electrolyte [18]. This last characteristic is important for biomedical applications, offering the possibility to create in one step the bioactive surfaces (containing for example Ca and P), which can promote cell adhesion/proliferation [19]. In particular, the formation of hydroxyapatite, the main inorganic compound of the bone, has to be favored because its presence on the surface of the implant can remarkably increase cell growth [20]. Due to the presence of the discharges, the surface of the PEO layers is rich in pores [21]. This aspect is very important for biomedical applications for two main reasons:

(a) the typical porous surface formed during the PEO treatment is ideal for cell growth and the pores can be filled with compounds that promote new tissues growth using proper post-treatments [22];

(b) although the coating reduces the sample’s corrosion rate, the presence of the pores implies that corrosion will proceed anyway, but more slowly than on the untreated alloy, permitting the degradation of the implant [23].

The production of PEO coatings on traditional magnesium alloy has been extensively studied in the literature [24,25]. Moreover, some important works can also be found regarding the study of PEO coatings on biodegradable and biocompatible magnesium. In particular, fluoride-containing coatings have been proved to increase the bioactivity of the sample and to delay the degradation for enough time to permit the bone repair [26,27,28,29,30]. The use of fluoride compounds is, however, not recommended due to environmental problems [31] and, indeed, only few works reported the formation in one step of hydroxyapatite-containing PEO coatings [32]. The surface composition and properties of hydroxyapatite depend on the production method, as evidenced by Skwarek et al. [33]. Therefore, the compound formed during the PEO process is worth investigating, considering that it could be different to the one obtained with other methods.

The aim of this work is to produce, by direct addition of Ca and P compounds in the electrolyte, a porous oxide ceramic coating containing hydroxyapatite on the surface of AZ31 magnesium alloy to control the degradation rate of AZ31, due to the increase in the corrosion properties. Since the porous nature on the coating, combined with the presence of hydroxyapatite, can favor, from literature data, both adhesion and proliferation of the cells, this work represents a preliminary step for further activities in view of a possible application of the coating in bioabsorbable implants. In comparison with the present literature, PEO coatings were produced working with short treatment times, and this can be very useful for technological applications. Moreover, no substances harmful for the environment were employed in the electrolyte.

## 2. Materials and Methods

AZ31 magnesium alloy (nominal composition (wt%) 3% Al, 0.9% Zn, 0.2% Mn, 0.1% Si, Mg balance (DSM, Beer-Sheva, Israel) was used as substrate for PEO treatment. This alloy is typically used for the studies in the biomedical field [34], even if some specific bioabsorbable magnesium alloys (mainly Mg-Ca) have been recently designed. Before PEO treatment, the AZ31 samples were polished by standard metallographic techniques (grinding with abrasive paper (Cloeren Technology, Padova, Italy) until 4000 grit and polishing with clothes (Cloeren Technology, Padova, Italy) and diamond suspension (Cloeren Technology, Padova, Italy) of 6 and 1 µm) and then degreased in ultrasonicated (ultrasonic bath, Branson Ultrasonics Corporation, Danbury, CT, USA) acetone (Sigma Aldrich, Milan, Italy). Two different aqueous electrolytes were employed to produce PEO coatings. The first electrolyte was a known solution, called standard, to produce anticorrosive coatings on magnesium alloys [35], with the composition 50 g/L Na_5_P_3_O_10_, 50 g/L Na_2_SiO_3_ and 40 g/L of NaOH (all the reagents are from Sigma Aldrich, Milan, Italy). The second electrolyte, called PEO-BIO, contained 1 g/L KOH, 10 g/L Na_5_P_3_O_10_, 8 g/L Na_2_SiO_3_ and 3 g/L of CaO (all the reagents were from Sigma Aldrich, Milan, Italy). This composition, starting from the literature, was selected, after optimization with preliminary tests, to favor the hydroxyapatite formation [19,23]. The purity grade of all the reagents employed in the production of the electrolytes for PEO process was between 96 and 100%. The plasma electrolytic oxidation process was carried out in Direct Current (DC) mode using a TDK-Lambda DC power supply (TDK-Lambda, Achem, Germany) of 400V/8A capacity. During the treatment, the sample worked as anode, and the cathode was a steel mesh (Agricola Cerchier, Eraclea, Italy). The electrolyte was magnetically stirred (magnetic stirrer, Velp Scientifica, Usmate Velate, Italy) during the treatment and maintained at room temperature by a cooling bath (Julabo, Seelbach, Germany). The PEO treatments were performed by maintaining a constant current of 0.5 A cm^−2^ and allowing voltage variations. The treatment last 90 s both for PEO and PEO-BIO samples. After the PEO process, the samples were washed with deionized water and ethanol (Sigma Aldrich, Milan, Italy) and dried with compressed air.

### 2.1. Microstructural Characterization

The microstructure, thickness, morphology and composition of the obtained coatings were analyzed by scanning electron microscopy (SEM) analysis and Energy Dispersive X-ray Spectroscopy (EDS) using a Cambridge Stereoscan 440 scanning electron microscope (Leica Microsystems S.r.l., Milan, Italy), equipped with a Philips PV9800 EDS (EDAX Inc Mahwah, NJ, USA). Both the surface and the cross sections were analyzed. In order to observe the cross sections, the samples were cut, mounted in epoxy resin (Cloeren Technology, Padova, Italy) and polished with a standard metallographic technique.

The phase composition of the PEO layers was evaluated by X-ray diffraction (XRD), performing θ–2θ scans from 20° to 90° with a 0.05 step size and a 5 s dwell time, by a Siemens D500 X-ray diffractometer (Siemens, Munich, Germany) with a Ni-filtered Cu-Kα radiation source (λ = 0.15405 nm), operating at 40 kV and 30 mA.

Surface composition was investigated by XPS measurements with a Φ 5600ci Perkin-Elmer spectrometer (Perkin Elmer, Milano, Italy), using a standard aluminum (Al Kα) source (energy of 1486.6 eV) operating at 200 W. The binding energy (B.E.) of the Au4f7/2 line at 83.9 eV with respect to the Fermi level was employed for calibration. The reported B.E.s were corrected for the B.E. charging effects, assigning a B.E. value of 284.6 eV to the C1s line of carbon. Survey scans were obtained in 0–1350 eV. Detailed scans were recorded for relevant regions (O1s, C1s, Al2p, Si2p, Na1s, Mg2s, Mg2p, Ca2p, Al2s, P2p, K2p). The atomic composition, after a Shirley-type background subtraction, was evaluated using sensitivity factors supplied by Perkin-Elmer. The samples were loaded onto the XPS sample holder by using conducting biadhesive tape. The acquired data were then interpreted with the use of the Multipak software package (Physical Electronics, Inc, Chanhassen, MN, USA). The assignments of the peaks were carried out by using the values reported in the reference handbook [36] and in the NIST XPS Database [37].

### 2.2. Corrosion Tests

The corrosion behaviors of the PEO and PEO-BIO treated samples were evaluated and compared with one of the untreated samples. The corrosion resistance was analyzed by potentiodynamic polarization (PDP) and electrochemical impedance spectroscopy (EIS) tests, in naturally aerated simulated body fluid (SBF), whose composition was 1.5881 g/L NaCl, 0.0709 g/L NaHCO_3_, 0.0492 g/L Na_2_HPO_4_·7H_2_O, 0.0617 g/L MgCl_2_·6H_2_O, 0.0746 g/L KCl, 0.0171 g/L CaSO_4_·H_2_O, and 0.0403 g/L CaCl_2_ (all the reagents are from Sigma Aldrich, Milan, Italy). The purity grade of all the reagents employed in the production of the electrolytes for corrosion tests was between 96 and 100%. Tests were performed at body temperature (37 ± 1 °C) using a thermostatic bath (Julabo, Seelbach, Germany) in order to reproduce human body conditions [38]. The choice of SBF as electrolyte for the corrosion tests was performed on the basis of the study of Mei et al. [39]. In fact, they state that for magnesium alloys used for bioabsorbable implants, synthetic pH buffers and nutrient-containing media should be avoided, using instead mild media such as SBF-like ones. They also state that the precise composition of SBF can significantly modify the results of the tests. In our case, the precise composition was decided on the basis of previous work of the authors [38]. The potentiodynamic tests were carried out after 1 h of stabilization at open circuit voltage (OCP) by an AMEL 2549 Potentiostat (Amel Electrochemistry S.r.l., Milan, Italy) using a saturated calomel electrode (SCE, Amel Electrochemistry S.r.l., Milan, Italy) as a reference electrode and a Pt counter electrode (Amel Electrochemistry S.r.l., Milan, Italy). The potential scans were performed at a scan rate of 0.4 mV s^−1^. The EIS measurements were performed in the previous described solution, at the value of the open circuit potential, after 1 h of stabilization, and in a frequency range between 10^5^ and 10^–2^ Hz with a perturbation amplitude of 10 mV. The electrochemical cell was the same of the PDP tests. The impedance measurements were recorded with a Materials Instrument Spectrometer (Amel Electrochemistry S.r.l., Milan, Italy) coupled with the 2549 Potentiostat and the ZView software (Scribner Associates Inc, Southern Pines, NC, USA) was used for the fitting of impedance spectra. All the electrochemical tests were performed in triplicate to assure reproducibility. In order to deeply study the behavior of the samples in an environment simulating the human body, long-duration immersion tests with weight-loss measurements were also performed. SBF solution at 37 °C was used as aggressive environment and the test lasted two months, with intermediate measurements at 1 week, 2 weeks, 1 month and 2 months. Three samples for each condition (untreated, PEO treated and PEO-BIO treated) were tested.

## 3. Results

### 3.1. Microstructure and Composition

**Figure 1:**
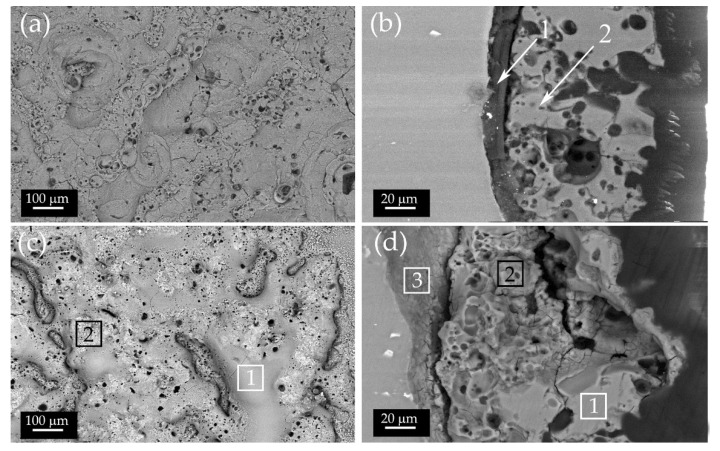
BSE-scanning electron microscopy (SEM) images of: (**a**) surface and (**b**) the cross section of the Plasma electrolytic oxidation (PEO) treated sample; (**c**) surface and (**d**) the cross section of the PEO-BIO treated sample. The arrows in (**b**) highlight the zones where Energy Dispersive X-ray Spectroscopy (EDS) analysis was performed in the cross section, whereas in (**a**) the analysis was performed on the whole surface. In (**c**), EDS analysis was performed on the whole surface and in the highlighted boxes, and in (**d**) this was performed in the highlighted boxes. Images of the surfaces were taken at 600×, whereas the ones of the cross section are at 1500×.

PEO and PEO-BIO samples were analyzed both on the surface and the cross section with SEM, and the results are reported in Figure 1. In both the samples, the surface (Figure 1a,c) was rich in pores, pancakes and nodular structures, the typical structures observed for PEO coatings [24]. It should be noted that pancake structures were more evident in the PEO-BIO sample. In the cross section of the PEO sample (Figure 1b), a typical double layer structure of PEO coatings was observed [25], with the inner barrier layer more compact (arrow 1) and an external porous layer (arrow 2). The thickness of the coating was about 90 µm in the PEO samples and variable between 90 and 120 µm in the PEO-BIO samples.

Observing the surface of the PEO-BIO sample (Figure 1c) in more detail, two different morphologies can be observed: a smooth zone, identified with number 1, and a rough and porous zone, identified by number 2. These differences in the morphology can also be found in the cross section (Figure 1d), where the two different zones are identified with the same numbers (1 and 2) and are located in the external porous layer. Additionally, in this sample the typical double layer structure of PEO coatings can be observed and the inner barrier layer is identified by number 3.

The results of the qualitative EDS analysis, performed both on the surface and the cross section in the different zones, are reported in Table 1. The surface in the PEO treated samples were mainly composed of Mg, Si, P, Na and O, in accordance with the composition of the substrate and of the electrolyte. The analysis carried out on the cross section showed that the external porous layer was characterized by a higher amount of silicon and sodium compared to the barrier layer. Considering the PEO-BIO sample, the presence of calcium-based compounds in the electrolyte led to the formation of a coating that contained Ca. From the extended analysis performed on the surface of the sample, the presence of both Ca and P was registered. In detail, magnesium was concentrated in the smooth zones (zone 1), whereas calcium and phosphorous were concentrated in the rough and porous zones (zone 2), with a Ca/P ratio of about 1, suggesting the formation of hydroxyapatite [40].

The analysis performed on the cross section showed a remarkable amount of Ca and P in the porous layer in comparison with the inner barrier layer, and a higher amount of Ca and P in the rough and porous zones was found (zone 2).

**Figure 2:**
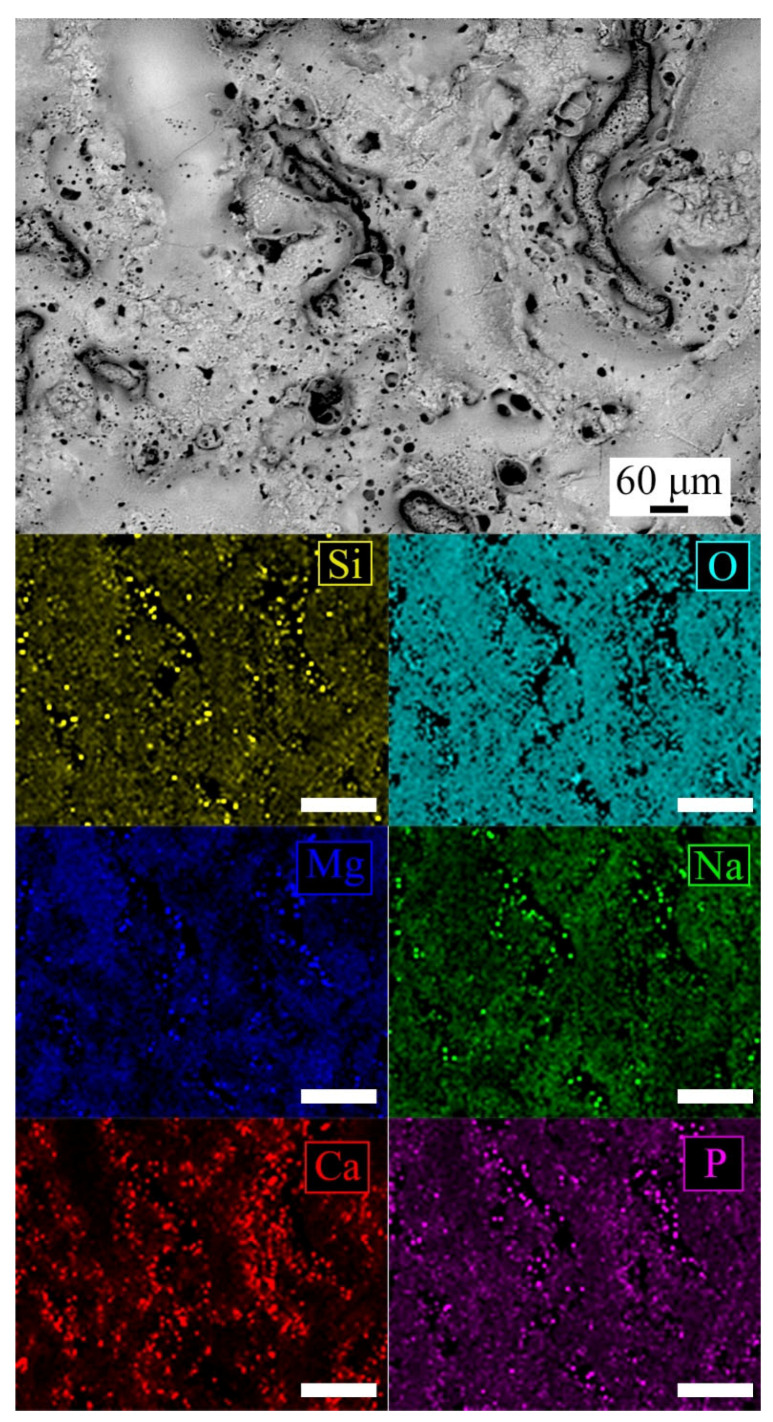
EDS elemental mapping, performed on the surface of the PEO-BIO sample. White lines in the images of the single elements represent 240 µm.

In order to confirm the distribution of the elements, EDS elemental mapping was performed on the surface of the PEO-BIO sample and the results are reported in Figure 2.

From observing of Figure 2, it can be seen that the smooth zones are mainly composed of Mg and Si oxides, whereas the rough and porous zones were rich in phosphorous and calcium.

**Figure 3:**
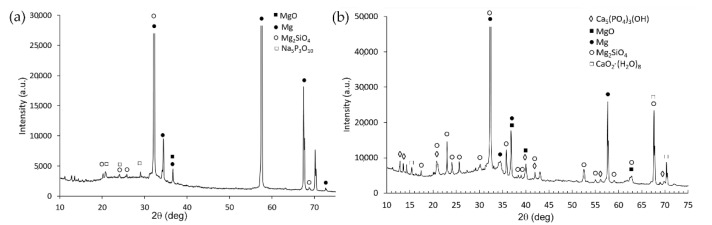
X-ray diffraction (XRD) pattern of the sample PEO treated (**a**) and PEO-BIO (**b**) samples.

In order to identify the phases of the PEO layers, and verify whether hydroxyapatite formed in the PEO-BIO sample, XRD analyses were performed on the PEO and PEO-BIO samples and the patterns are reported in Figure 3a,b. In both the samples peaks of Mg can be observed due to the reflection from the substrate. Considering the PEO treated sample, the protective oxide film was composed mainly of MgO and Mg_2_SiO_4_, with also the presence of sodium compounds coming from the electrolyte. Considering the PEO-BIO sample, in addition to MgO and Mg_2_SiO_4_, calcium-containing phases were observed. In detail, CaO_2_(H_2_O)_8_ and Ca_5_(PO_4_)_3_(OH) (hydroxyapatite) were detected. The presence of hydroxyapatite plays a key role in the biocompatibility of the samples as the osteoblast adhesion was extremely favored by this phase [20]. Considering the previous results of EDS, the hydroxyapatite was probably concentrated in the more porous zones, and this can further favor cell adhesion.

**Figure 4:**
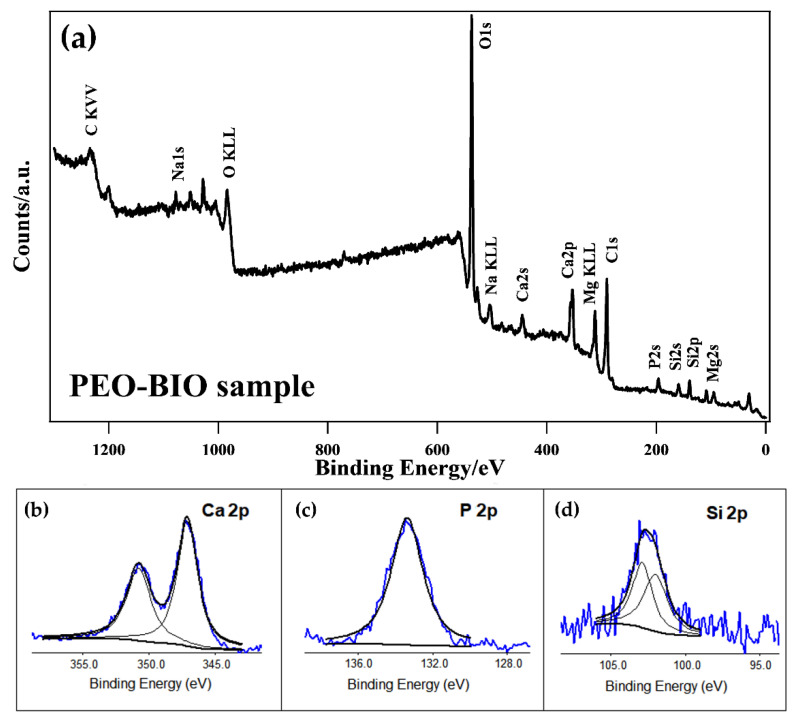
XPS survey scan (**a**) and XPS high-resolution single peak spectra of the Ca2p region (**b**), the P2p region (**c**) and the Si2p region (**d**) for the PEO-BIO sample.

The PEO-BIO sample was also analyzed with XPS in order to determine the chemical nature of the elements constituting the surface, their relative amounts and their oxidation state. A survey scan is shown in Figure 4a, whereas the detailed composition of the surface of the samples is shown in Table 2. The recorded elements were O, C, Mg, Ca, Si, P and Na. The main componentof the surface was oxygen (about 43% at.), due to the oxidative PEO process, and carbon, due to the surface contamination.

From high-resolution analysis of the Ca2p peak (Figure 4b), the Ca2p spectrum was found to be constituted of two peaks: one at 347.1 eV for Ca2p3/2 and one at 350.7 eV for Ca2p1/2. The Ca2p peaks in the XPS spectra were attributed to the presence of Ca_3_(PO_4_)_2_ and Ca_10_(PO_4_)_6_(OH)_2_ [41].

The P2p spectrum showed one peak at 133.3 eV (Figure 4c), suggesting the presence of PO_4_^3−^ in the coating [41].

The high-resolution Si2p peak, reported in Figure 4d, is the sum of two peaks. The peak situated at 101.9 eV Binding Energy (B.E.) was attributed to α-Mg_2_SiO_4_, whereas the one at 102.9 eV B.E. corresponded to γ-Mg_2_SiO_4_ [42].

From literature data, both the porous nature of PEO coatings and the presence of hydroxyapatite can favor cell adhesion and proliferation. In detail, Robinson et al. [22] evidenced that the adhesion strength of the cells on PEO layers produced on titanium was stronger than the one on the base metal; Santos-Coquillat et al. [43] proved, both from in vitro and in vivo tests, that the cell proliferation on PEO coated titanium was higher than the one on the uncoated metal. Clearly, further studies must be performed on Mg alloys in order to prove, with both in vitro and in vivo tests, the improved performances of the PEO-BIO sample in terms of cell adhesion and proliferation.

### 3.2. Corrosion Resistance

**Figure 5:**
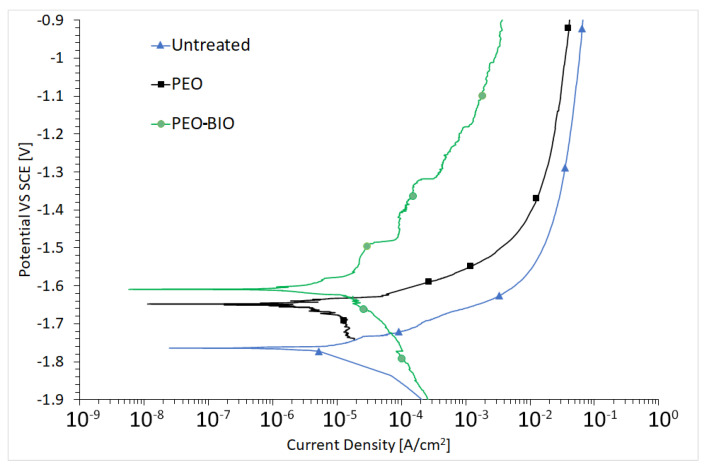
Potentiodynamic polarization plot of the different samples. Test electrolyte: simulated body fluid at 37 °C.

Considering that corrosion is one of the main issues in the employment of magnesium alloys as potential bioabsorbable material, the corrosion resistance of the various samples was analyzed with both electrochemical techniques and immersion tests. The results of PDP tests are reported in Figure 5. From the graphs, an ennoblement of corrosion potential was observed for the coated specimens, if compared with the untreated one, with a decrease in the corrosion current for PEO and PEO-BIO samples. The results of PEO-BIO samples are promising; the objective is to slow down the degradation of the alloy but not to prevent it, so that the prostheses can be reabsorbed in an acceptable period of time once their purpose has been fulfilled. Moreover, the release of bioactive molecules, due to the degradation of the substrate, is fundamental during the healing process [19].

**Figure 6:**
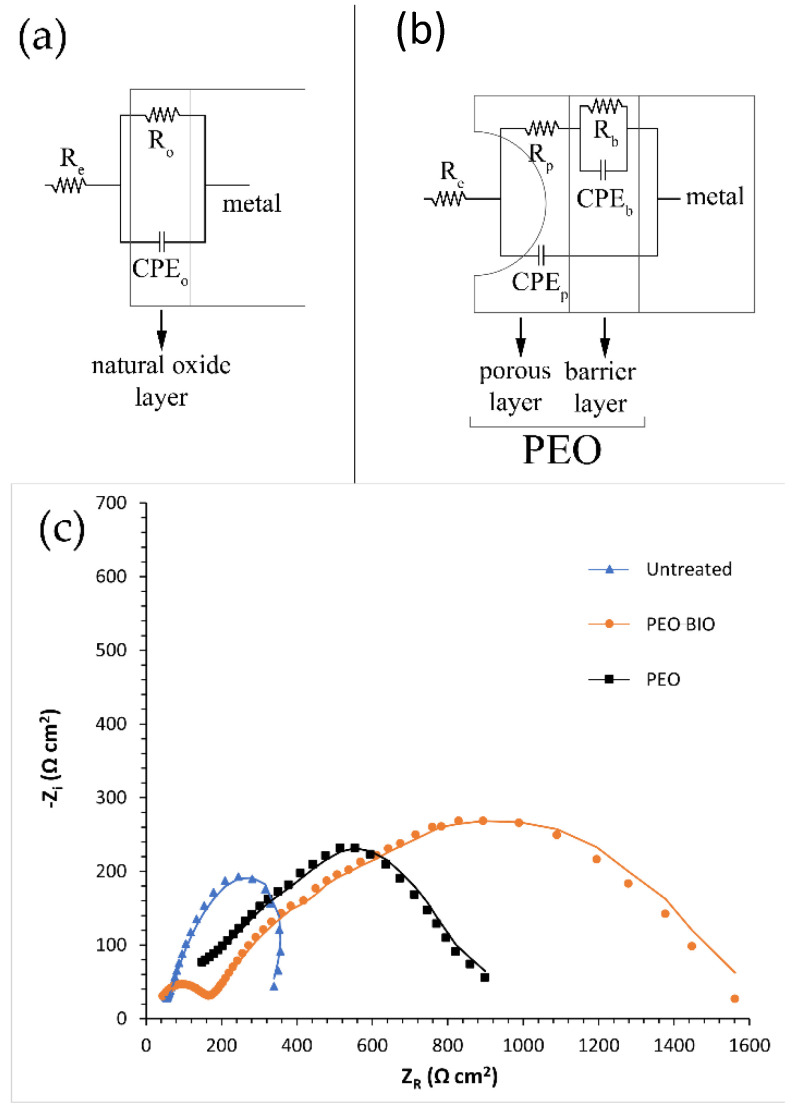
Equivalent circuits employed to fit the electrochemical impedance spectroscopy (EIS) experimental data coming from the untreated sample (**a**) and the PEO and PEO-BIO samples (**b**) and Nyquist plot coming from EIS tests performed on the different samples (**c**). Dots represent experimental data and lines are the results of the fitting. Test electrolyte: simulated body fluid at 37 °C.

Potentiodynamic polarization tests were performed for a qualitative and comparative analysis due to the fact that on samples coated with a thick insulating oxide layer, the Tafel law cannot be applied and so corrosion current densities and corrosion potentials cannot be calculated [44]. For quantitative analysis, electrochemical impedance spectroscopy (EIS) tests were performed. The fitting of the experimental data was performed with Z-view software, using the equivalent circuits reported in Figure 6a,b. Two different equivalent circuits were employed to consider the presence of different protective layers in the various samples. For the untreated sample, a simple Resistance-Constant Phase Element (R/CPE) circuit was employed as only a natural oxide layer is present (Figure 6a), whereas the equivalent circuit of Figure 6b was used for the data coming from PEO coated and PEO-BIO samples. This type of circuit is the one often employed in literature to fit data from PEO samples [35], in order to consider the presence of two layers: an external porous layer and an internal layer, often called a barrier layer.

In these equivalent circuits, R_e_ represents the resistance of the electrolyte, R_p_ the polarization resistance of the porous layer and R_b_ the polarization resistance of the barrier layer. In the untreated sample, the polarization resistance of the natural oxide layer is called R_o_. This schematization was employed in order to consider the formation of two different electrolyte-substrate interfaces. The first interface is the one between the electrolyte and the external porous layer, whereas the second is the one between the electrolyte in the pores and the internal barrier layer. Instead of capacitances, CPEi were used in the equivalent circuit to consider the fact that the measured capacitance is not ideal. The results of the EIS tests are reported in terms of a Nyquist plot in Figure 6c and the results of the fitting of the experimental data are presented in Table 3. Good quality of the fitting can be observed, and in fact both the low values of chi-squared and the good correspondence between dot and lines indicate the obtainment of a good fit. Comparing the behavior of the different samples, an increase in the corrosion resistance was observed for both PEO and PEO-BIO samples in comparison with the untreated one. Considering the contribution of the different layers for both PEO coated samples, the polarization resistance of the inner barrier layer was increased compared to the one of the external porous one, in agreement with the absence of a sealing treatment. The PEO-BIO sample was characterized by a slightly higher corrosion resistance than the PEO one, and this can be related to the thicker layer in the PEO-BIO sample that increases the barrier effect.

**Figure 7:**
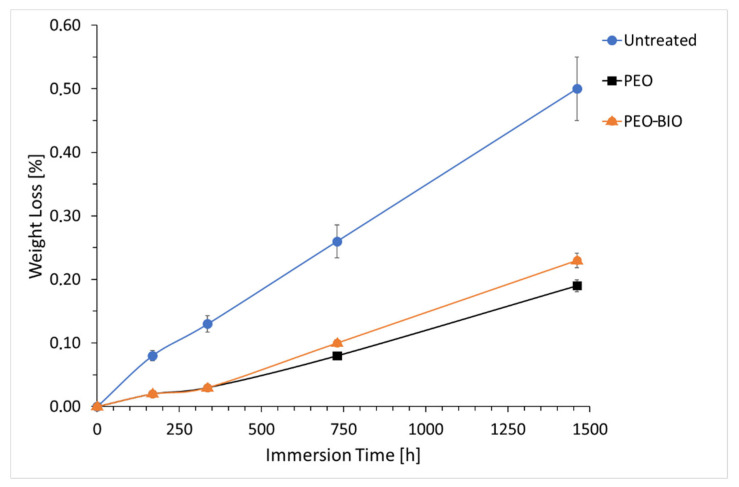
Results of the weight loss measurements of the different samples. Test electrolyte: simulated body fluid at 37 °C.

In order to better understand the corrosion behavior in an environment that simulates the human body, also long-duration immersion tests were performed in SBF at 37 °C with weight loss measurements. The analysis lasted two months and the results are reported in Figure 7. The increased corrosion performances of the PEO coated samples in comparison with the untreated one were confirmed, and this can be considered positive for implant application, as the healing process will have time to take place before the degradation of the PEO-BIO coated device occurs.

For the first two weeks of immersion, the PEO and PEO-BIO samples showed the same behavior. After two weeks, the PEO-BIO sample started to corrode faster than the PEO one, even if the differences in the corrosion rates were quite small. Additionally, this fact can be considered positive considering that the final objective is the degradation of the PEO-BIO coated device in the human body. The results of the immersion test evidenced a different behavior of the two PEO treated samples in comparison with the EIS test. This fact can be related to the type of test: the immersion test is less affected by the thickness of the coatings in comparison with EIS and depends more on the capacity of the electrolyte to penetrate into the coating to reach the substrate.

Further studies to evaluate the in vivo corrosion performances must be performed, because, as can be found in the literature [45], the correlation between in vitro and in vivo corrosion performances is not always reliable.

## 4. Conclusions

In this preliminary work, PEO coatings containing hydroxyapatite, that according to literature data can favor osteointegration, were produced on an AZ31 magnesium alloy. The presence of hydroxyapatite, verified by both XPS and XRD analyses, was obtained thanks to the direct addition of Ca and P compounds to the electrolyte and to the interactions between the electrolyte, the substrate and the discharge phenomena. The obtained coatings were characterized in terms of microstructure and compared with a standard PEO coating, evidencing, for the PEO-BIO sample, an increase in the thickness and in the pancake structures on the surface. A gradient in the composition was observed with calcium-rich compounds present mainly on the external porous layer. Both PEO coated samples showed improved corrosion performances in comparison with the untreated one, and the PEO-BIO sample showed a similar behavior to the standard one. The presence of hydroxyapatite and the porous nature of the coating, which from the literature can promote both cell growth and adhesion, and the improved corrosion performances, which can allow the healing process to take place, suggest that the proposed treatment can be promising for an eventual application in bioabsorbable implants, even if further in vitro and in vivo studies have to be performed.

## Figures and Tables

**Table 1:** Qualitative EDS analysis (wt%) of the surface and of the cross section of the PEO and PEO-BIO samples.

Sample	Mg%	Al%	O%	Si%	Na%	P%	Ca%
PEO Surface	19.7	1.0	40.2	16.3	18.9	3.9	-
PEO Barrier Layer (1)	48.9	1.3	34.1	8.7	4.4	2.6	-
PEO Porous Layer (2)	17.7	0.6	38.8	20.1	17.1	5.7	-
PEO-BIO Surface (Extended analysis)	17.9	0.8	41.4	9.4	8.1	11.9	10.5
PEO-BIO Surface (1)	32.4	-	50.7	4.8	2.9	6.3	2.9
PEO-BIO Surface (2)	10.7	-	48.9	8.5	7.7	12.5	10.8
PEO-BIO Barrier Layer (3)	50.0	2.0	34.7	4.4	2.5	4.7	1.7
PEO-BIO Porous Layer/Rough Zone (2)	24.8	1.1	31.4	14.0	2.0	13.7	13.0
PEO-BIO Porous Layer/Smooth Zone (2)	38.9	0.8	37.3	10.4	1.6	6.3	4.7

**Table 2:** Surface composition (atomic %) of the PEO-BIO sample, recorded with XPS analysis.

Sample	C%	O%	Mg%	Ca%	Si%	P%	Na%
PEO-BIO	36.9	42.9	6.3	5.2	4.0	3.8	0.9

**Table 3:** Results of the fitting of the experimental data coming from EIS tests.

Sample	R_e_ [Ω cm^2^]	R_o_ and R_b_ [Ω cm^2^]	R_p_ [Ω cm^2^]	Q_o_ and Q_b_ [F cm^−2^ Hz^1−n^]	n_o_ and n_b_	Q_p_ [F cm^−2^ Hz^1−n^]	n_p_	χ^2^
Untreated	44	462	-	2.05 × 10^−5^	0.7	-	-	0.002
PEO	45	480	257	3.91 × 10^−6^	0.86	1.56 × 10^−6^	0.74	0.003
PEO-BIO	45	877	103	9.10 × 10^−4^	0.55	1.59 × 10^−6^	0.93	0.005

## Data Availability

The raw/processed data required to reproduce these findings cannot be shared at this time as the data also forms part of an ongoing study.
